# A heparin-binding protein of *Plasmodium berghei* is associated with merozoite invasion of erythrocytes

**DOI:** 10.1186/s13071-023-05896-w

**Published:** 2023-08-10

**Authors:** Junying Gao, Ning Jiang, Yiwei Zhang, Ran Chen, Ying Feng, Xiaoyu Sang, Qijun Chen

**Affiliations:** 1 Key Laboratory of Livestock Infectious Diseases, Ministry of Education, Key Laboratory of Zoonosis, College of Animal Science and Veterinary Medicine, Shenyang Agricultural University, 120 Dongling Road, Shenyang, 110866 China; 2 Research Unit for Pathogenic Mechanisms of Zoonotic Parasites, Chinese Academy of Medical Sciences, 120 Dongling Road, Shenyang, 110866 China

**Keywords:** *Plasmodium*, Heparin-binding protein, Invasion, Pathogenicity

## Abstract

**Background:**

Malaria caused by *Plasmodium* species is a prominent public health concern worldwide, and the infection of a malarial parasite is transmitted to humans through the saliva of female *Anopheles* mosquitoes. *Plasmodium* invasion is a rapid and complex process. A critical step in the blood-stage infection of malarial parasites is the adhesion of merozoites to red blood cells (RBCs), which involves interactions between parasite ligands and receptors. The present study aimed to investigate a previously uncharacterized protein, *Pb*MAP1 (encoded by PBANKA_1425900), which facilitates *Plasmodium berghei* ANKA (*Pb*ANKA) merozoite attachment and invasion via the heparan sulfate receptor.

**Methods:**

*Pb*MAP1 protein expression was investigated at the asexual blood stage, and its specific binding activity to both heparan sulfate and RBCs was analyzed using western blotting, immunofluorescence, and flow cytometry. Furthermore, a *Pb*MAP1-knockout parasitic strain was established using the double-crossover method to investigate its pathogenicity in mice.

**Results:**

The *Pb*MAP1 protein, primarily localized to the *P. berghei* membrane at the merozoite stage, is involved in binding to heparan sulfate-like receptor on RBC surface of during merozoite invasion. Furthermore, mice immunized with the *Pb*MAP1 protein or passively immunized with sera from *Pb*MAP1-immunized mice exhibited increased immunity against lethal challenge. The *Pb*MAP1-knockout parasite exhibited reduced pathogenicity.

**Conclusions:**

*Pb*MAP1 is involved in the binding of *P. berghei* to heparan sulfate-like receptors on RBC surface during merozoite invasion.

**Supplementary Information:**

The online version contains supplementary material available at 10.1186/s13071-023-05896-w.

## Background

Malaria remains one of the world’s most important fatal diseases, with at least 241 million malaria cases and 627,000 malaria deaths recorded worldwide each year [1]. The growth and replication of *Plasmodium* parasites in red blood cells (RBCs) and the rupture of infected RBCs (iRBCs) are responsible for most symptoms of malaria [2]. Therefore, the blood stage of the parasites is the primary target for intervention. The process of erythrocyte invasion by the merozoites involves four continuous steps: attachment of a merozoite to the erythrocyte surface, followed by apical reorientation, tight junction formation, and final invasion [3]. As the adhesion of a merozoite to the RBC surface is essential to complete invasion, it is logical to believe that molecules presented on the merozoite surface play a critical role in this process. To date, several merozoite surface proteins as well as rhoptry- and microneme-derived proteins have been shown to play roles in the invasion process [4]. However, many proteins are associated with erythrocyte invasion, the functions of which remain to be explored further.

Heparan sulfate (HS), a non-branched polysaccharide of glycosaminoglycans (GAGs), which are widely distributed on the surface of vertebrate cells, is utilized by *Apicomplexan* parasites to invade host cells [5–11]. The adhesion of *Toxoplasma gondii* surface antigens and secreted proteins to host cells via heparin/HS has been reported. Specifically, *T. gondii* invasion-related proteins, such as surface protein 1 (SAG1), rhoptry protein 2 (ROP2), ROP4, and dense granule antigen 2 (GRA2), bind heparin [12]. HS is an essential receptor for *Plasmodium falciparum* that recognizes erythrocytes prior to invasion. HS also functions as a receptor for *P. falciparum* erythrocyte membrane protein 1 (*Pf*EMP1), a parasite virulent factor associated with rosette formation that can adhere to normal erythrocytes [13, 14]. In addition, *P. falciparum* merozoite surface protein 1 (*Pf*MSP-1), reticulocyte-binding homolog (*Pf*RH5), and erythrocyte-binding protein (*Pf*EBA-140) have been reported to bind to HS on the surface of RBCs and are disrupted by heparin [11, 15, 16]. Additionally, HS inhibits the invasion of erythrocytes by *Plasmodium berghei* [17]. However, many proteins expressed on the *P. berghei* merozoite surface that interact with HS remain poorly characterized.

In the present study, we identified a novel protein (*Pb*MAP1) on the surface of *P. berghei* that binds heparin and HS. The specificity of *Pb*MAP1 binding to HS on the surface of RBCs was characterized. *Pb*MAP1-specific antibodies inhibited parasite invasion. Parasites with gene deletion exhibited reduced pathogenicity in mice. Our findings suggest that *Pb*MAP1 plays a critical role in merozoite invasion of erythrocytes.

## Methods

### Animals

Female BALB/c mice, aged 6–8 weeks, were purchased from Liaoning Changsheng Biological Technology Company (Benxi, China) and maintained in an animal laboratory using a standard protocol. All animal experiments were conducted in accordance with the institutional guidelines of the Shenyang Agricultural University, China (ethical approval no. SYXK < Liao > 2021-0010).

### Parasite proliferation

Parasites were revitalized from frozen infected RBC stocks and maintained by infecting BALB/c mice through intraperitoneal (IP) injection of 1 × 10^6^ parasitised erythrocytes per mouse, as previously described [18]. Peripheral blood parasitemia was assessed using Giemsa-stained thin blood smears.

### Bioinformatic analysis and gene identification

To identify genes encoding potential merozoite surface proteins, we searched the PlasmoDB malaria database for proteins expressed in merozoites with a putative signal peptide or an external or at least one transmembrane domain [19]. We identified PBANKA_1425900, which encodes a putative protein of 154 kDa, named *Pb*MAP1. *Pb*MAP1 (PBANKA_1425900) and orthologous sequences in *Plasmodium yoelii* (PY17X_1428000), *P. chabaudi* (PCHAS_1427700), *P. vinckei* (PVSEL_1402900), *P. falciparum* (PF3D7_0811600), *P. ovale* (PocGH01_14036000), *P. malariae* (PMALA_030180), *P. gaboni* (PGSY75_0811600), and *P. reichenowi* (PRCDC_0810900) were obtained by searching within the PlasmoDB database. Sequence similarities were determined using DNAMAN 6 (Lynnon Corp., USA).

### Generation of recombinant *Pb*MAP1 protein

The gene encoding *Pb*MAP1 was cloned using RT-PCR with total RNA isolated from *P. berghei*-infected erythrocytes. The amplified product was cloned into the pGEX-4T-1 expression vector. The GST-tagged recombinant protein was expressed in BL21 (DE3) cells, and the soluble protein was purified using Glutathione Sepharose (GE Healthcare) via affinity purification.

### Heparin-binding and competition assays

Purified GST-*Pb*MAP1 and GST proteins (5 nmol) were mixed with 40 μl heparin-Sepharose beads and incubated at 4 °C for 2 h. The beads were washed seven times with phosphate buffer saline (PBS) and centrifuged at 500×g for 5 min at 4 °C. The pellets were boiled in 1× SDS-PAGE loading buffer to analyze the heparin-binding activity by western blotting. Sepharose was used as the control.

To analyze the specific affinity between the protein and heparin, 5 nmol GST-*Pb*MAP1 was incubated with 50 μl heparin or chondroitin sulfate A (CSA, Sigma Aldrich, USA) at various concentrations (10, 1, 0.1, 0.01, 0.001, and 0.0001 mg ml^-1^) for 30 min at 4 °C. Subsequently, 40 μl heparin-Sepharose beads were gradually added to the mixture and incubated for 2 h at 4 °C. Finally, the heparin-Sepharose beads were washed seven times with PBS and centrifuged at 500×g for 5 min at 4 °C. The effect of competition was analyzed using western blotting. Heparin-binding and competition assays were conducted as previously described [20].

### Binding of recombinant *Pb*MAP1 to RBCs

Affinity-purified GST-*Pb*MAP1 and the GST control protein were respectively incubated with mouse RBCs (7 μl packed volume) in PBS (pH 7.4) for 1 h at 37 ℃ (final volume 1 ml) [21]. The RBC pellet was washed seven times in 1 ml PBS, and proteins bound to the RBCs were analyzed with western blotting using GST mAb (1:3000; TransGen).

To further confirm the binding of *Pb*MAP1 to mouse RBC, freshly collected RBCs in PBS were washed, resuspended in RPMI (20% hematocrit), and fixed on glass slides with methanol (20–30 s) as a thin smear. The fixed RBCs were washed in PBS, blocked with 3% bovine serum albumin (BSA) for 1 h at 37 °C, washed again (5 times, 10 min each), and incubated with 5 nmol GST-*Pb*MAP1 or 5 nmol GST in 3% BSA for 2 h at 37 °C. Slide samples were washed five times with PBS, incubated with goat anti-GST antibody (1:3000; TransGen), washed five times, and incubated with Alexa Fluor 488 goat anti-mouse IgG (H+L) (1:500; Sigma). High-resolution images were captured using a fluorescence microscope (Leica Microsystems). The mean fluorescence intensity of the RBCs was determined using the FACSAria III flow cytometer (BD Biosciences).

### Generation of *Pb*MAP1-specific antibodies

A peptide (CLYKSFKFVPPKKKLTDYFEEMFSDHKVEYDSLENVSKQL) from the *Pb*MAP1 protein was synthesized by Sangon Biotech. Rats and mice were immunized to obtain polyclonal antibodies. For the first immunization, 50 μg peptide per mouse emulsified with complete Freund’s adjuvant was subcutaneously injected, followed by three immunizations with 50 μg peptide per mouse emulsified with incomplete Freund’s adjuvant at 2-week inter-injection intervals. The mice in the control group were injected with Freund’s adjuvant. Antibody titers were evaluated using ELISA. Briefly, to coat microplates with recombinant *Pb*MAP1, 50 μl of the prepared working *Pb*MAP1 protein solution was added to each well of a 96-well ELISA plate (0.5 μg *Pb*MAP1/well). The plate was incubated overnight at 4 ℃. Subsequently, the unbound coating *Pb*MAP1 solution was discarded, and the wells were washed three times with 200 μl PBST. The remaining protein-binding sites in the coated wells were blocked by adding 50 μl of 3% BSA per well of the microtiter plate and incubated for 1 h at 37 ℃. Next, after washing three times, the plate was incubated with 50 μl diluted serum samples (1:500, 1:1000, 1:2000, 1:4000, 1:8000, 1:16,000, and 1:32,000) at 37 ℃ for 1 h for binding reaction, followed by 50 μl HRP anti-mouse IgG antibodies in the dark at 37 ℃ for 1 h. The unbound HRP anti-mouse IgG antibodies were washed five times with 200 μl PBST. For substrate reaction, 100 μl of 3,3′,5,5′-tetramethylbenzidine (TMB) was added to each well and incubated in the dark at 37 ℃ for 15 min for blue color development. At the next stage, 50 μl of 2M H_2_SO_4_ was added to each well to quench the reaction. Absorbance was read with a microtiter plate reader at 450 nm immediately after the addition of 2M H_2_SO_4_. The antibodies for the samples were determined using the following formula: titer = (optical density [OD] values of sample—OD values of blank control)/(OD values of negative control—OD values of blank control).

### Indirect immunofluorescence assay (IFA) to localize the expression of *Pb*MAP1

Infected red blood cell smears were fixed in methanol at room temperature for 10 min. The cells were marked with a hydrophobic PAP pen and washed five times with PBS. The cells were blocked with 3% BSA. Primary anti-*Pb*MAP1 antibodies (1:50) were diluted in 3% BSA and added to the slides. The cells were incubated overnight at 4°C and subsequently washed five times with PBS. Next, secondary anti-rat Alexa Fluor 488 antibodies (1:500) (Thermo Fisher Scientific) were added to the slides. The cells were incubated for 30 min at 37 °C and then washed five times with PBS. The cells were mounted with ProLong Diamond Antifade Mountant with DAPI (Thermo Fisher Scientific) and analyzed under a fluorescence microscope (Leica).

### Passive immunization assays with *Pb*MAP1-immune sera

In passive immunization assays, each mouse was intravenously (IV) injected with 500 μl sera from peptide-immunized mice 24 h earlier. After 24 h of challenging with 1 × 10^6^ iRBCs, each mouse was IV injected with 500 μl sera from peptide-immunized mice again. Parasitemia was measured and counted as described above.

### Construction of plasmids for *Pb*MAP1 gene editing

We used the double-crossover method to generate a *Pb*MAP1-knockout strain, as previously described [22]. The plasmid was constructed using pl0001 (MR4; ATCC, USA). *Pb*MAP1 5′- and 3′-untranslated regions (UTRs) were PCR-amplified as the left or right homologous arms and inserted respectively upstream and downstream of the DHFR gene. The 5′-UTR and 3′-UTR were subcloned in the pl0001 plasmid using restriction enzyme sites (*Apa* I/*Bsp*D I or *Bam*H I/*Not* I).

To re-introduce the *Pb*MAP1 gene into the knockout strain (Δ*Pb*MAP1), the CRISPR/Cas9 plasmid pYCm (a gift from Prof. Jing Yuan, Xiamen University) was used, and a parasitic strain with a reconstructed *Pb*MAP1 gene was generated in Δ*Pb*MAP1 strain. Oligonucleotides for guide RNA (sgRNA) were designed using the Eukaryotic Pathogen CRISPR guide RNA/DNA Design Tool (http://grna.ctegd.uga.edu/) and ligated into the pYCm. Mutagenesis was performed using the Q5 Site-Directed Mutagenesis Kit (NEB, E0552). Specifically, the 5′-UTR and 3′-UTR (400 to 700 bp) of the target genes were amplified as left or right homologous arms using specific primers (Additional file 1: Table S1) and inserted into the restriction sites in pYCm.

### Transfection of *Pb*ANKA parasites

*Plasmodium berghei* was transfected as previously described [22]. Two BALB/c mice were injected intraperitoneally with erythrocytes from *P. berghei*-infected mice (1-4 droplets of 5%–15% parasitemia). Once parasitemia ranged between 1% and 3%, the parasites were collected and cultured in vitro to mature schizonts in RPMI 1640 containing 20% FBS. A total of 5 μg linear plasmid (digested with *Kpn* I and *Sac* II) was mixed with 1 × 10^7^ schizonts purified with 60% Nycodenz (AXIS-SHIELD). The mixture was transferred to an electroporation cuvette and pulsed at 800 V and 25 μF capacitance using Gene Pulser II (Bio-Rad). To the transfected parasites, 100 μl of complete culture medium was added immediately, and the suspension was IV injected into another BALB/c mouse. The mice were administered drinking water containing pyrimethamine (70 μg ml^-1^) 1 day after infection with transfected parasites (with knockout plasmids). Pyrimethamine was administered for 4–9 days up to the collection of infected blood. In addition, a single dose of 0.1 ml WR99210 (16 mg kg^-1^ body weight in mice weighing 20 g) was administered 3 days after infection with transfected parasites (with complemented plasmid). Genomic DNA was extracted from parasites for PCR analysis. Parasite clones with the correct modifications were obtained using the limiting dilution method. Positive clones were tested using PCR, western blotting and IFA. The primers used for genotyping are listed in Additional file 1: Table S1.

### Confirmation of *Pb*MAP1 expression in gene-edited parasites with southern blotting

Southern blotting of *Pb*MAP1 and Δ*Pb*MAP1 parasites was performed using the DIG High Prime DNA Labelling and Detection Starter Kit (Roche) according to the product manual. Briefly, genomic DNA was digested by Hind III for 1 h at 37 °C and separated on a 0.8% agarose gel for southern blotting onto a Hybond N^+^ nylon transfer membrane (Millipore). The target gDNA bands were hybridized with a digoxigenin-labeled DNA probe complementary to *Pb*MAP1 and detected using an anti-digoxigenin AP-conjugated antibody. The primers used for probe amplification are listed in Additional file 1: Table S1.

### Infectivity tests of mice with wild-type *P. berghei* and gene-edited strains

For comparison the infectivity of wild-type *P. berghei* and gene-edited strains, 90 female BALB/c mice were randomly divided into nine groups of 10 mice each and were intraperitoneally administered with specific doses (1 × 10^3^, 1 × 10^4^, or 1 × 10^6^) of *P. berghei* wild-type (WT), Δ*Pb*MAP1 (knockout strain), or ReΔ*Pb*MAP1 (the gene re-complement strain) strains. Parasitemia was assessed daily by counting cells in Giemsa-stained smears from a droplet of tail blood. Animals were monitored daily for survival.

### RNA extraction and quantitative real-time PCR (qRT-PCR)

The parasites were harvested from the blood of BALB/c mice infected with WT or Δ*Pb*MAP1 strains. Total RNA was extracted [23] from the parasite pellets using the TRIzol reagent (Invitrogen), dissolved in RNase-free pure water, and quantified using a NanoDrop spectrophotometer (Nanodrop 2000/2000c). First-strand cDNA was synthesized from random primer mixes using PrimeScript RT Enzyme Mix I (Takara), according to the manufacturer’s instructions. Specific primers (Additional file 1: Table S1) were designed to detect the mRNA transcription of certain genes after *Pb*MAP1 knockout. The cycling conditions were as follows: initial denaturation at 95 °C for 30 s, followed by 40 cycles of denaturation at 95 °C for 5 s, annealing at 60 °C for 30 s, and extension at 72 °C for 30 s. The housekeeping gene actin (PBANKA_1459300) was used to normalize the transcriptional level of each gene.

### Serum cytokine analyses

Levels of IFN-γ, TNF-α, MCP-1, CCL4, IL-6, IL-10, and IL-12p70 in mouse sera were determined using the Cytometric Bead Array Mouse Inflammation kit (BD Biosciences). Samples were assayed according to the standard protocol (BD Biosciences) and examined using the FACSAria III flow cytometer (BD Biosciences) driven by the FACSDiva software (BD Biosciences).

## Results

### The sequence of *Pb*MAP1 is conserved across *Plasmodium* species

**Fig. 1:**
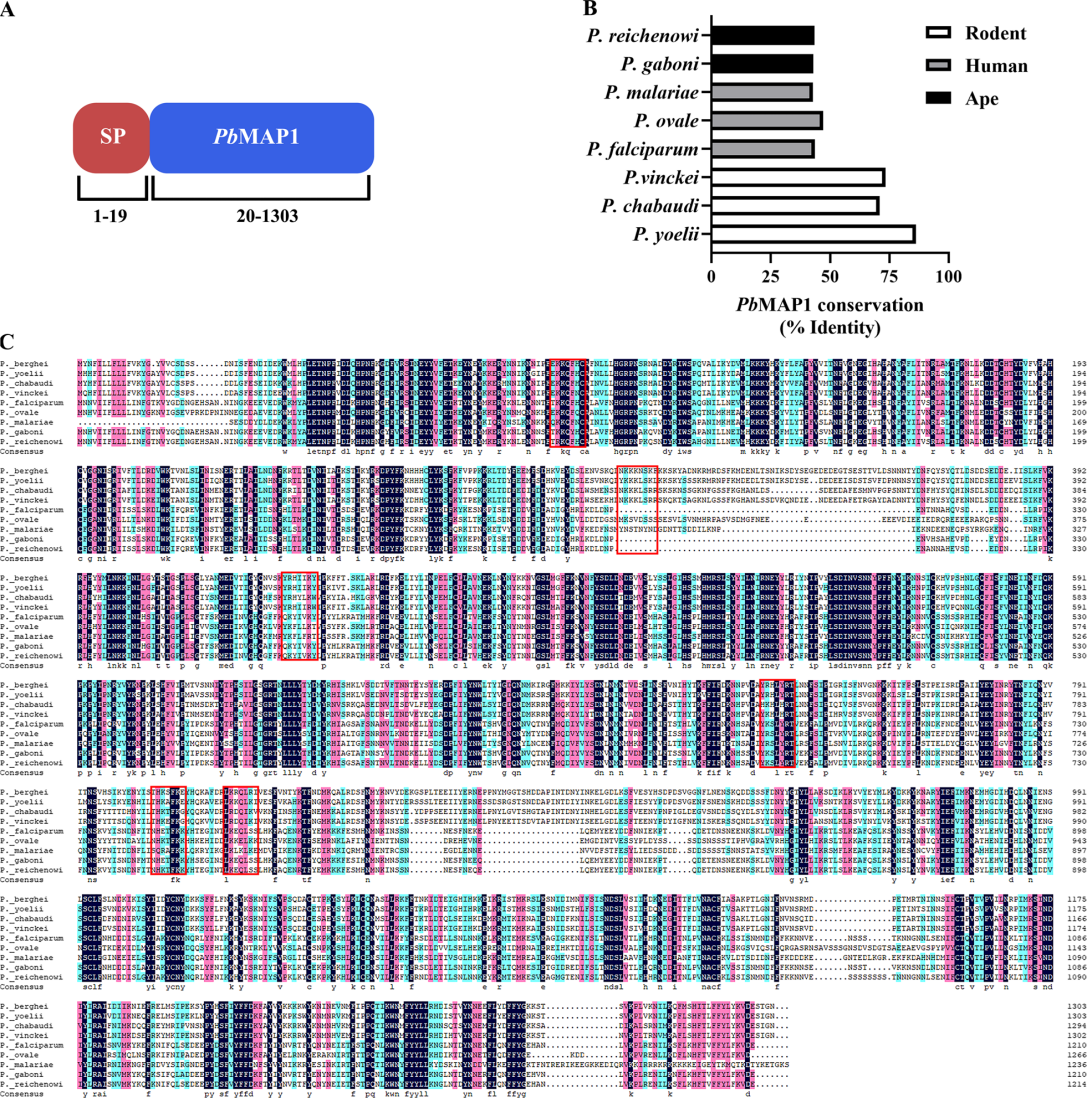
Sequence analysis of *Pb*MAP1. **A** *Pb*MAP1 is 1303-amino acid long and predicted to contain a signal peptide. **B** Identity in amino acid sequence between *Pb*MAP1 and orthologous sequences from other *Plasmodium* species. **C** Alignment of amino acid sequence of *Pb*MAP1 with orthologs of *Plasmodium* species (sequence identities are listed in the Methods section). The putative heparin binding motifs in the sequences are highlighted in the red box

The gene coding for *Pb*MAP1 is located on chromosome 14 and encodes a protein of 1303 amino acids, which is predicted to contain a signal peptide at the N-terminus (Fig. 1A). The amino acid sequence of *Pb*MAP1 is relatively conserved across *Plasmodium* species, with > 70% identity among *P. yoelii*, *P. chabaudi*, and P. *vinckei* orthologs, > 40% identity across species infecting humans (including the zoonotic *P. knowlesi*), and > 43% identity with species infecting apes (Fig. 1B, 1C).

### *Pb*MAP1 specifically binds heparin

**Fig. 2:**
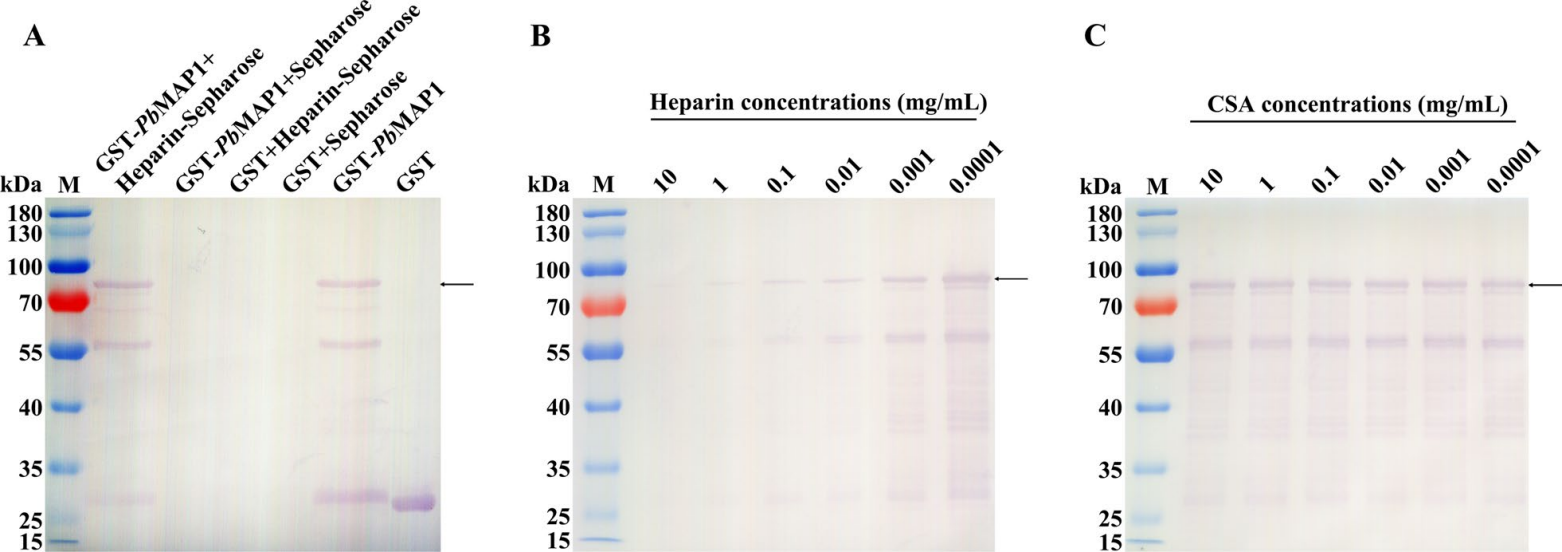
GST-*Pb*MAP1 specifically bound to heparin. **A** Heparin-binding activity was detected using western blotting with anti-GST antibody. GST-*Pb*MAP1 could bind heparin-Sepharose but not Sepharose. GST could not bind heparin-Sepharose. **B** Competitive inhibition was detected using western blotting with an anti-GST antibody. As the heparin concentration increased, GST-*Pb*MAP1 binding with heparin-Sepharose gradually decreased. **C** As the CSA concentration increased, no significant changes in GST-*Pb*MAP1 binding with heparin-Sepharose were noted

To demonstrate the properties of *Pb*MAP1 binding to heparin, the recombinant protein GST-*Pb*MAP1 was purified. GST-*Pb*MAP1 could bind to heparin-Sepharose but not to Sepharose, whereas GST could not bind to heparin (Fig. 2A). The competitive inhibition assay revealed that with an increase in heparin concentration, the binding amount of GST-*Pb*MAP1 to heparin decreased gradually (Fig. 2B); however, no significant change in the binding of GST-*Pb*MAP1 to heparin was noted with increase in CSA concentration (Fig. 2C).

### Recombinant *Pb*MAP1 could specifically bind to mouse RBCs

**Fig. 3:**
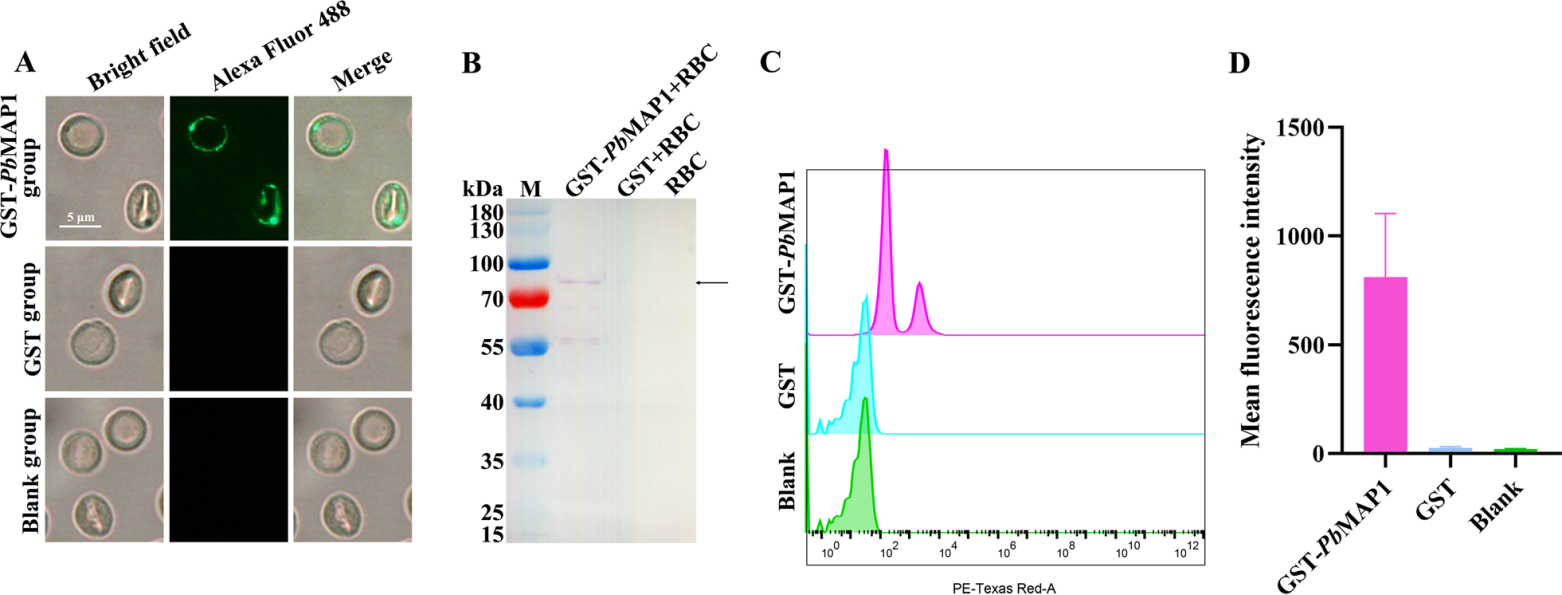
GST-*Pb*MAP1 binds to mouse RBCs. **A** Indirect immunofluorescence assay using an anti-GST antibody as the primary antibody and Alexa Fluor 488 Goat anti-mouse IgG (H+L) as the secondary antibody shows the binding of GST-*Pb*MAP1 to RBCs. GST was used as the control. Scale bar, 5 μm. **B** Western blotting using an anti-GST antibody shows the binding of GST-*Pb*MAP1 to RBCs. **C** Cell flow cytometry analysis of erythrocyte binding by the GST-*Pb*MAP1 with GST as a control. A clear shift is observed with GST-*Pb*MAP1-bound erythrocytes. **D** The fluorescence intensity of erythrocytes bound with GST- *Pb*MAP1 compared to that with GST and blank controls

GST-*Pb*MAP1 and GST were incubated with RBCs and analyzed using IFA, western blotting, and flow cytometry. IFA showed that the RBCs incubated with GST-*Pb*MAP1 exhibited specific green fluorescence on the surface, whereas the RBCs incubated with GST alone showed no fluorescence (Fig. 3A). Western blotting showed that specific target bands were detected for RBCs incubated with GST-*Pb*MAP1, while no bands were detected for RBCs incubated with GST alone (Fig. 3B). The binding of GST-*Pb*MAP1 to RBCs was analyzed using flow cytometry, and the results confirmed that *Pb*MAP1 could bind to RBCs (Fig. 3C). Therefore, *Pb*MAP1 could bind to the surface of RBCs.

### *Pb*MAP1 localization on merozoites

**Fig. 4:**
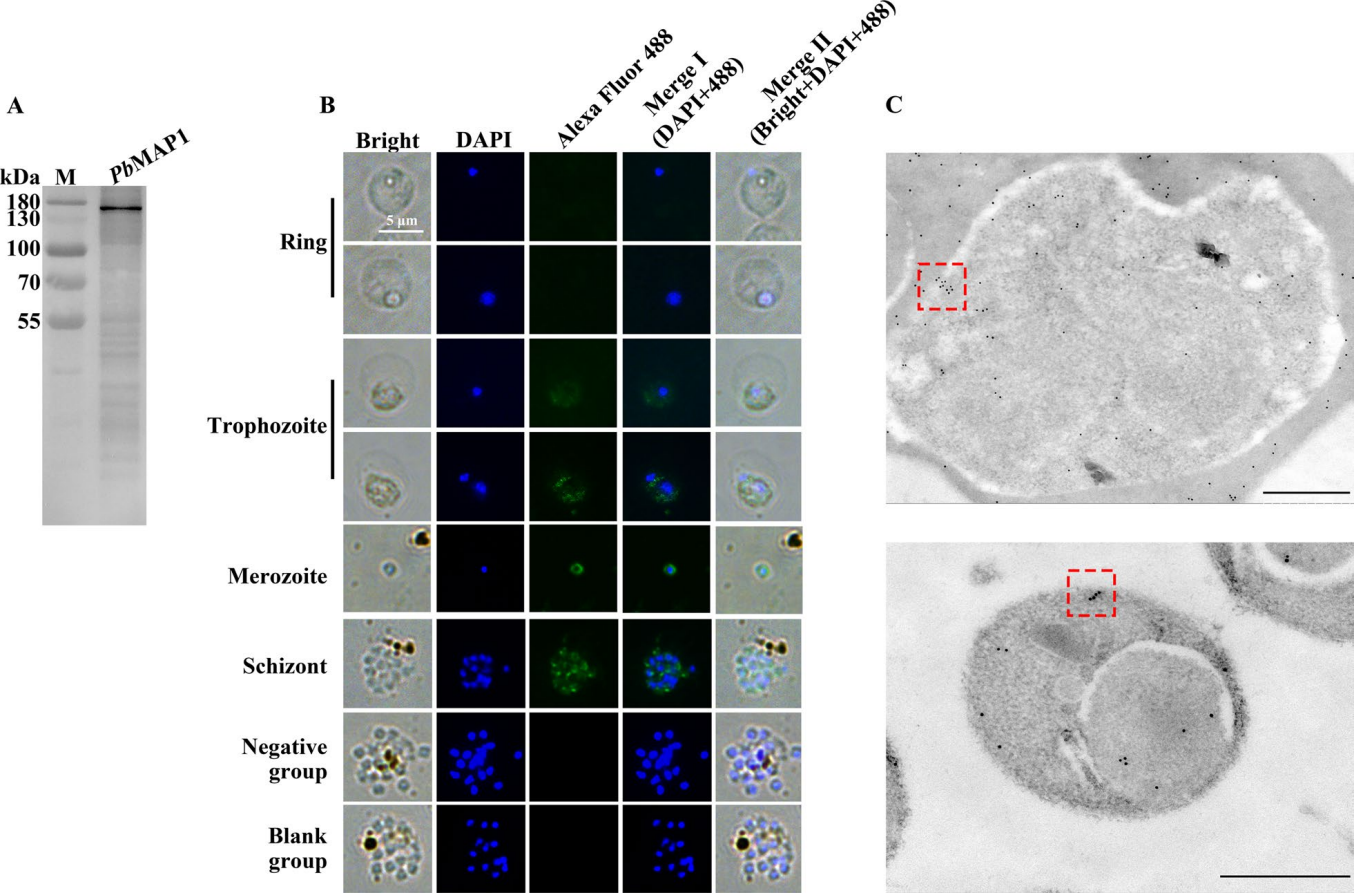
*Pb*MAP1 is expressed in merozoites. **A** Western blotting of native *Pb*MAP1 expressed in *Plasmodium berghei* merozoite detected using *Pb*MAP1-specific antibodies. **B** Indirect immunofluorescence of *Pb*MAP1. Free merozoites and parasite at ring-, trophozoite-, and schizont stages were fixed, and *Pb*MAP1 expression was detected with anti-*Pb*MAP1 IgG (green). Parasite nuclei were stained with DAPI (blue). **C** Immune electron microscopy images of *Pb*MAP1. The gold particles were localized to the merozoite membrane. Scale bar, 500 nm

To investigate the localization of *Pb*MAP1 in merozoites, western blotting, IFA, and immunoelectron microscopy were performed using *Pb*MAP1-specific antibodies. Western blotting confirmed *Pb*MAP1 expression in merozoites. Next, we analyzed the expression and localization of *Pb*MAP1 using IFA. Fluorescence was observed on the surface on free merozoites and that in the schizonts. Immunoelectron microscopy further verified that *Pb*MAP1 was mainly located on the plasma membrane (Fig. 4A–C).

### Immunization with *Pb*MAP1-specific peptides protects against infection

**Fig. 5:**
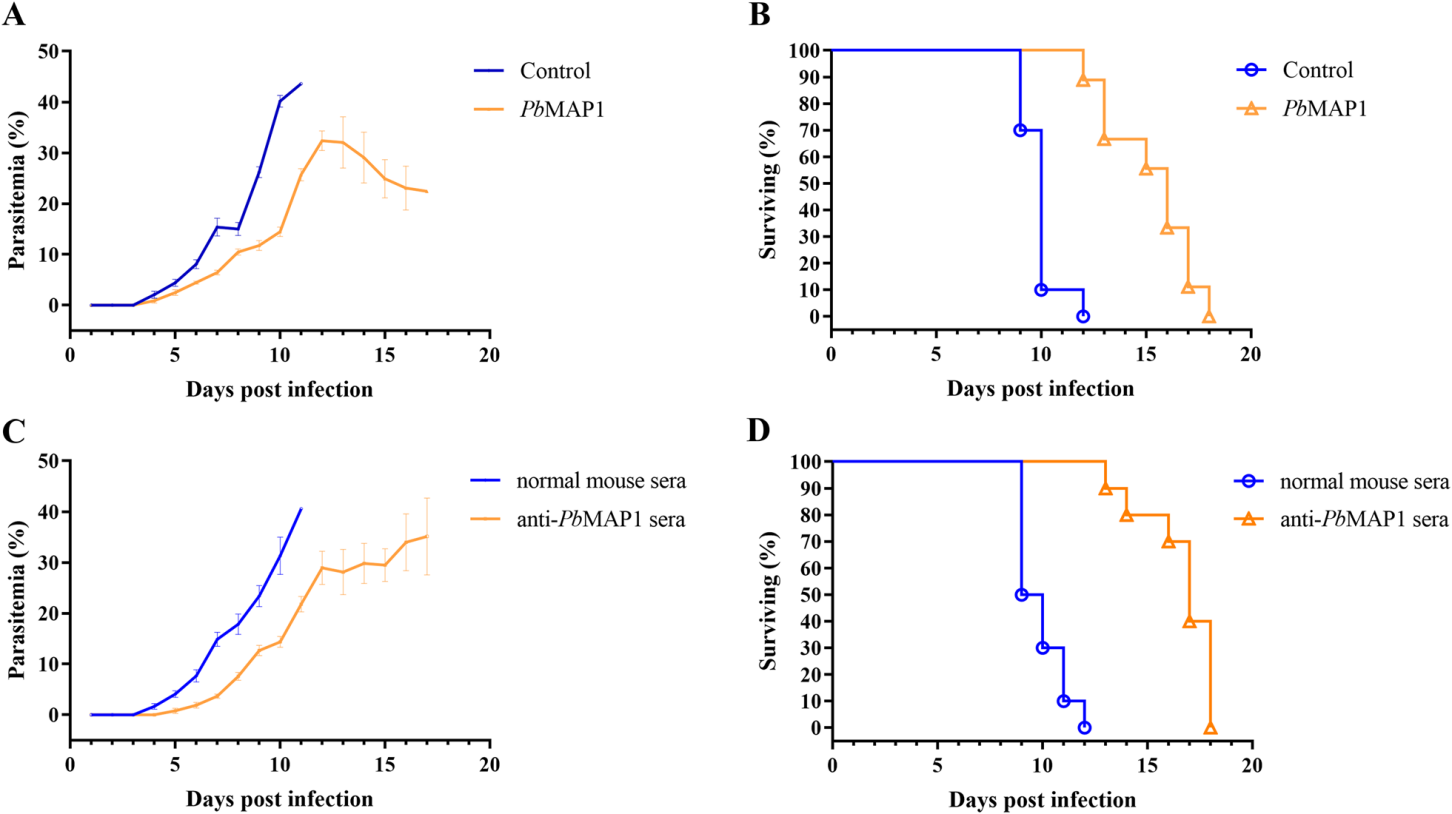
*Pb*MAP1-specific antibodies generated protective immunity against *Plasmodium berghei* ANKA. **A**, **B** BALB/c mice without any immunization (control) exhibited 1.70-fold higher parasitemia than *Pb*MAP1 peptide-immunized mice on day 11 post-infection; the error bars indicate SD. Mice immunized with *Pb*MAP1 peptides survived 6 days longer than control mice. **C**, **D** Mice injected with serum from a normal infected mouse exhibited 1.86-fold higher parasitemia than those injected with anti-*Pb*MAP1 sera on day 11 post-infection. Compared with the normal mouse serum group, the anti-*Pb*MAP1 serum group survived for 6 days longer; the error bars indicate SD

In this assay, we aimed to determine whether *Pb*MAP1-specific antibodies protected the host from parasitic infections in vivo. BALB/c mice (*N* = 10 per group) were immunized four times with specific peptides. The mice with high antibody titers were injected with 1 × 10^6^ iRBCs, and their survival time and parasitemia were measured. The mice immunized with specific peptides showed significantly longer survival (Fig. 5A, B). Furthermore, hyperimmune serum was collected and intravenously injected into BALB/c mice, followed by the injection of 1 × 10^6^ iRBCs. The survival of the mice injected with anti-*Pb*MAP1 serum was significantly longer than that of the control mice. Therefore, *Pb*MAP1 antibodies could inhibit the invasion of parasites and produce immune-protective effects (Fig. 5C, D).

**Establishment of a PbMAP1**-**knockout (ΔPbMAP1) strain**

**Fig. 6:**
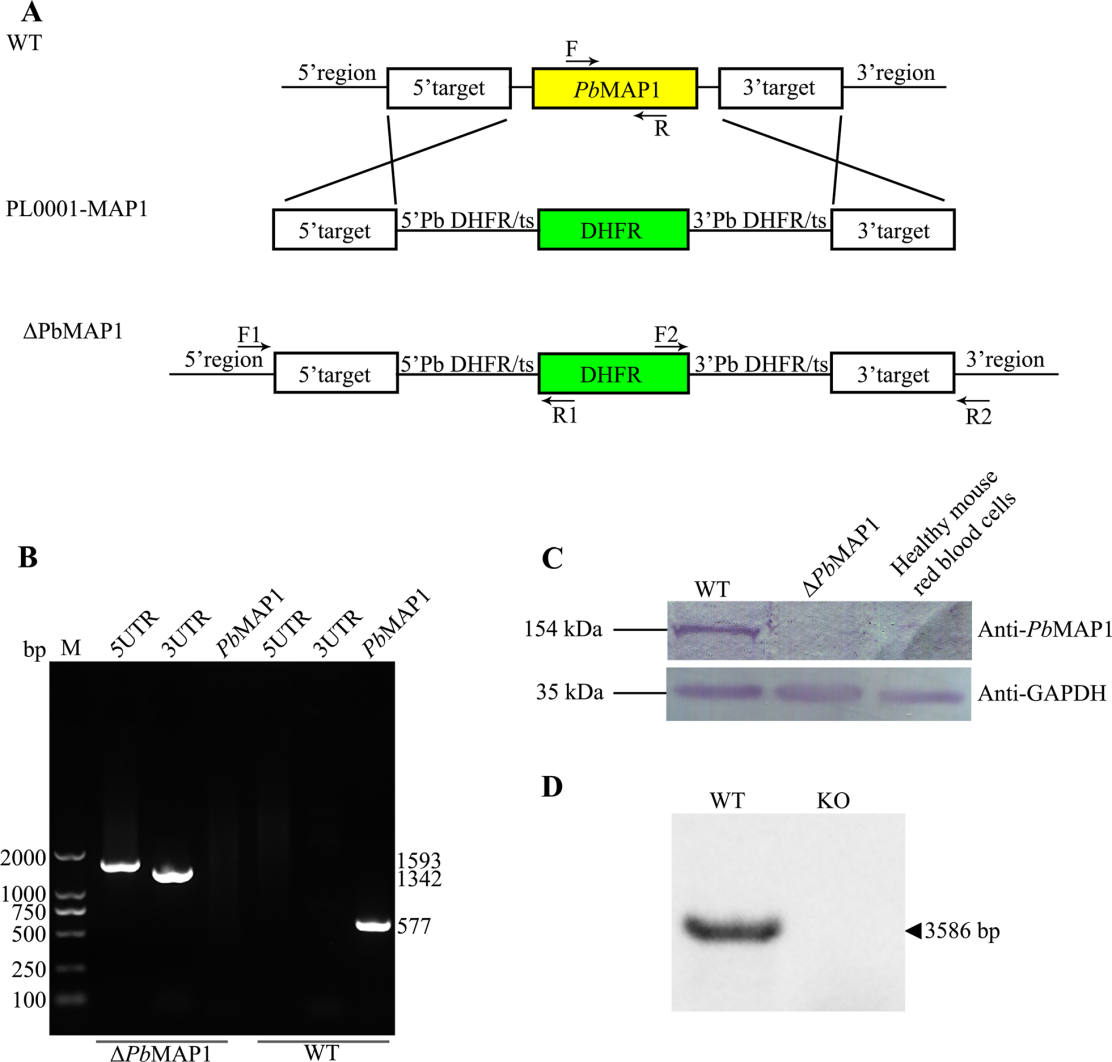
Construction of a *Pb*MAP1-knockout strain (Δ*Pb*MAP1). **A** Scheme of the transfection plasmid used to target and knock out *Pb*MAP1 in the *Pb*ANKA strain using the double-crossover method. **B** PCR validation of *Pb*MAP1 deletion. PCR products from 5'-UTR, 3′-UTR, and *Pb*MAP1 showed that *Pb*MAP1 was replaced by DHFR. **C** Western blotting of *Pb*MAP1 expression in WT and Δ*Pb*MAP1. Protein bands with expected molecular weights are shown. No *Pb*MAP1 protein was detected in the knockout strain. GAPDH was used as the control. **D** Southern blotting using a DNA probe from the *Pb*MAP1 gene in WT and Δ*Pb*MAP1. The gene encoding *Pb*MAP1 was completely deleted in the knockout strain

We examined whether *Pb*MAP1 is a key factor for binding to the surface of RBCs by knocking out the *Pb*MAP1 gene from the parasite (Fig. 6A). The knockout strain (Δ*Pb*MAP1) was cloned and verified using PCR with specific primers (Fig. 6B). Moreover, western blotting confirmed virtually complete deletion of *Pb*MAP1 (Fig. 6C), and no signal was detectable in southern blotting in the Δ*Pb*MAP1 strain (Fig. 6D).

### Construction of the *Pb*MAP1 gene re-complement (ReΔ*Pb*MAP1) strain

**Fig. 7:**
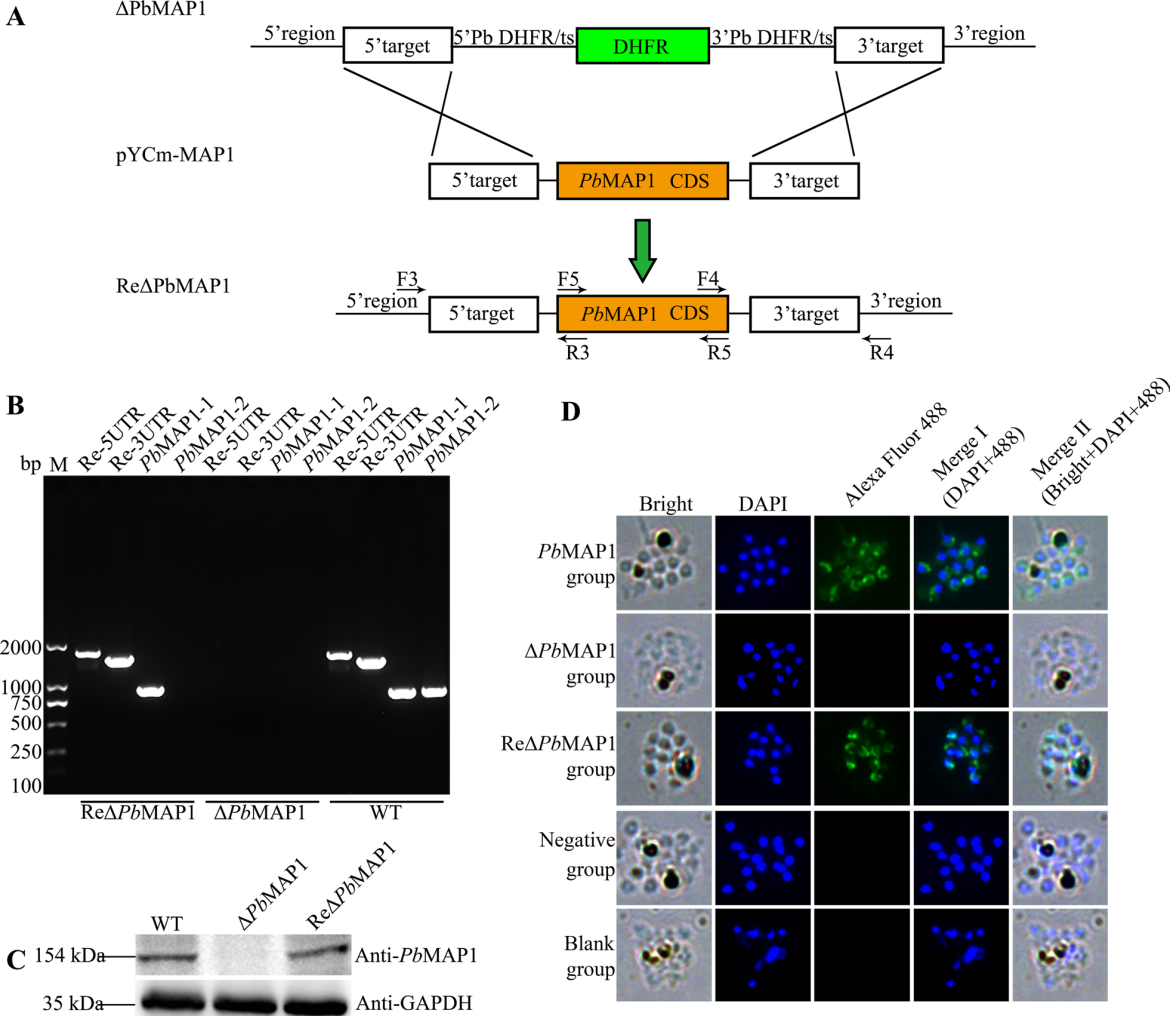
Construction of the *Pb*MAP1-complemented strain (ReΔ*Pb*MAP1). **A** Scheme of the transfection plasmid used to target and re-introduce *Pb*MAP1 gene in Δ*Pb*MAP1 using the CRISPR/Cas9 method. **B** PCR validation of *Pb*MAP1 reintroduction. PCR products from 5′-UTR, 3′-UTR, and *Pb*MAP1 showed that *Pb*MAP1 was re-introduced. **C** Western blotting of *Pb*MAP1 expression in Δ*Pb*MAP1 and ReΔ*Pb*MAP1. No protein band with expected molecular weight was detected in the Δ*Pb*MAP1 strain, but in the ReΔ*Pb*MAP1 strain; GAPDH was used as the control. **D** IFA using rat anti-*Pb*MAP1 IgG to detect *Pb*MAP1 expression. *Pb*MAP1 was detected in WT and ReΔ*Pb*MAP1 but not in the Δ*Pb*MAP1 strain

To demonstrate that the phenotype defect was due to the deletion of the gene coding for *Pb*MAP1, we re-introduced the *Pb*MAP1 gene without intron at the endogenous *Pb*MAP1 locus in the Δ*Pb*MAP1 parasite (Fig. 7A). The gene complemented parasite (ReΔ*Pb*MAP1) was cloned and verified using PCR with specific primers (Fig. 7B). Western blotting and IFA also confirmed *Pb*MAP1 expression in the ReΔ*Pb*MAP1 strain (Fig. 7C, D).

### Deletion of *Pb*MAP1 led to virulence deficiency

**Fig. 8:**
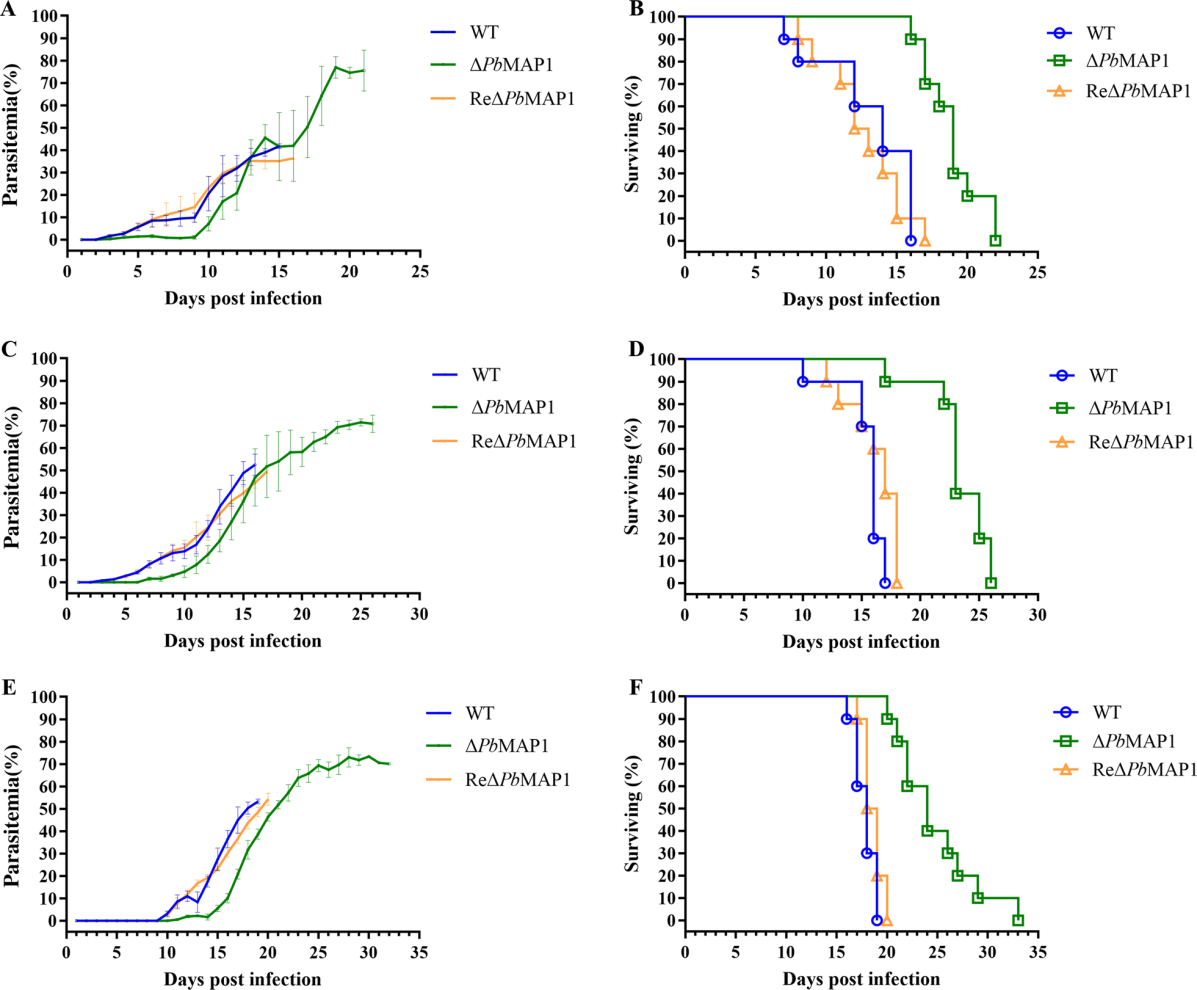
*Pb*MAP1 deletion reduced the infectivity of *Pb*MAP1. **A** and **B** Parasitemia and survival curves of BALB/c mice after intraperitoneal infection with 1 × 10^6^ WT, Δ*Pb*MAP1, or ReΔ*Pb*MAP1 parasites. **C** and **D** Parasitemia and survival curves of BALB/c mice after intraperitoneal infection with 1 × 10^4^ WT, Δ*Pb*MAP1, or ReΔ*Pb*MAP1 parasites. **E** and **F** Parasitemia in and survival curves of BALB/c mice after intraperitoneal infection with 1 × 10^3^ WT, Δ*Pb*MAP1, or ReΔ*Pb*MAP1 parasites.

To investigate the correlation of the *Pb*MAP1 with parasite infectivity, we infected mice with WT *Pb*MAP1, Δ*Pb*MAP1, or ReΔ*Pb*MAP1 at 1 × 10^6^ parasites per mouse. Tail blood was analyzed daily post-infection using Giemsa-stained smears, which revealed that the degree of parasitemia in mice infected with the Δ*Pb*MAP1 parasites was lower than that in mice infected with WT parasites. Moreover, the survival time of these mice was monitored. Mice infected with Δ*Pb*MAP1 parasites died 4–10 days later than those infected with WT. All mice died of severe anemia (Fig. 8A, B). A similar phenomenon was noted in mice infected with WT, Δ*Pb*MAP1, or ReΔ*Pb*MAP1 when infected with 1 × 10^3^ or 1 × 10^4^ parasites per mouse (Fig. 8C–F).

**Fig. 9:**
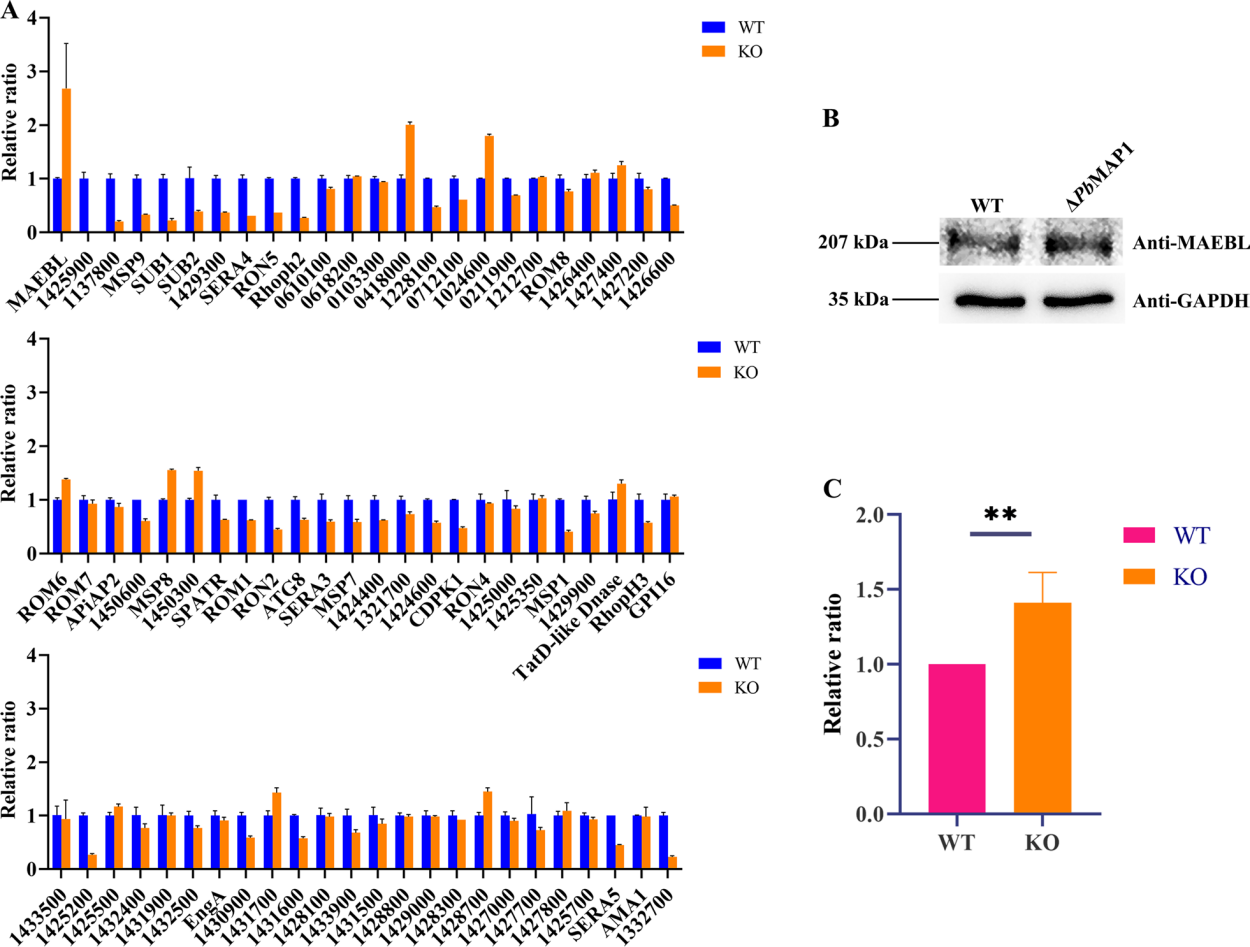
Transcription analysis of invasion-related genes in the Δ*Pb*MAP1 parasites with qRT-PCR. **A** Transcription of *MAEBL* was significantly upregulated in Δ*Pb*MAP1 strains compared to that in the wild-type strain. **B** and **C** Expression of MAEBL protein in Δ*Pb*MAP1 strains was positively correlated with transcription levels. Experiments were repeated three times. Error bars represent SD

Interestingly, as the parasites continued to divide and proliferate, their virulence appeared to recover. To confirm that the phenomenon of virulence recovery was the result of *Pb*MAP1 knockout, qRT-PCR was performed on specific genes in the WT and Δ*Pb*MAP1 parasites (Fig. 9A). Compared with WT, *Pb*MAP1-knockout strains showed significantly upregulated transcript levels of *MAEBL* gene coding for the merozoite adhesive erythrocytic binding protein (MAEBL), and significantly downregulated transcript levels of 10 other genes, suggesting a complementary association between *Pb*MAP1 and MAEBL. Western blotting showed that the expression level of the MAEBL protein in Δ*Pb*MAP1 strains was higher than that in WT strains (Fig. 9B, C).

### Infection with Δ*Pb*MAP1 induced stronger IFN-γ and TNF-α responses in infected mice

**Fig. 10:**
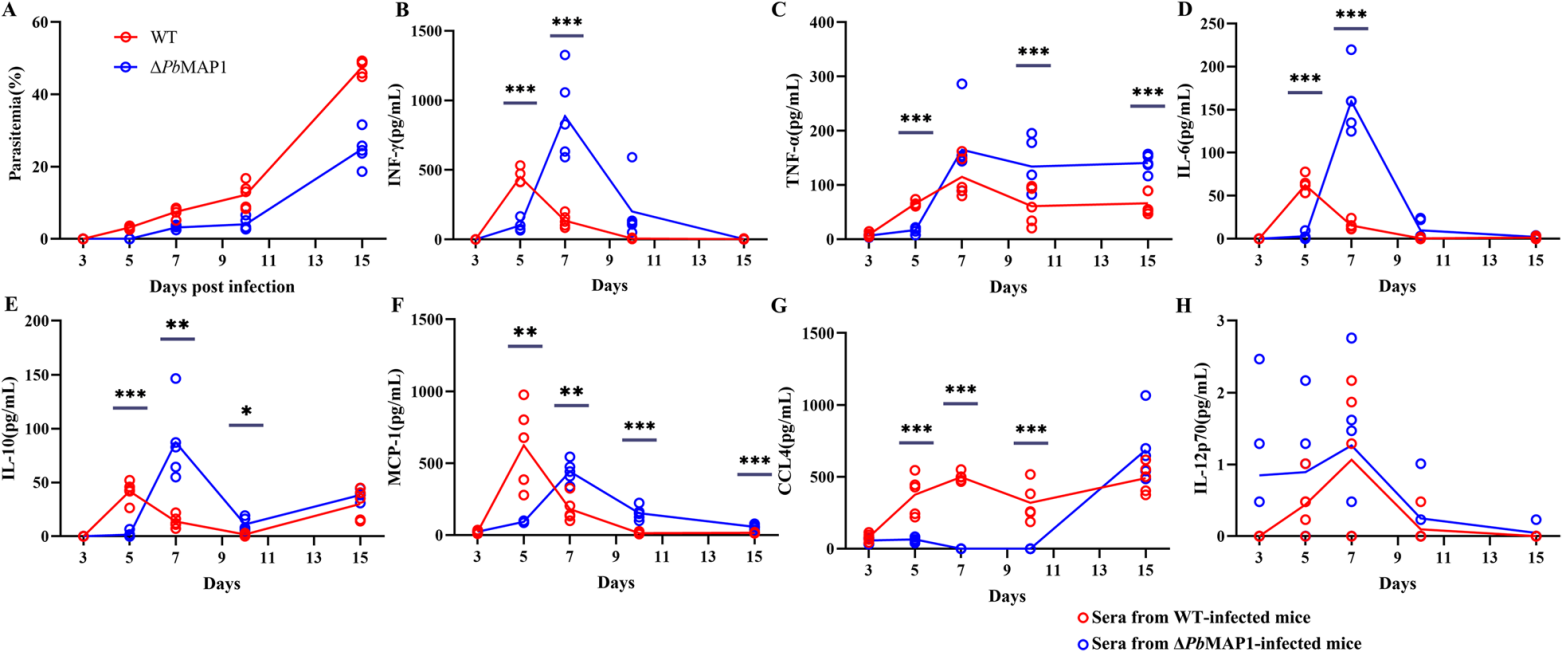
Δ*Pb*MAP1 infection induced stronger IFN-γ and TNF-α responses in infected mice. **A** BALB/c mice were infected with either Δ*Pb*MAP1 or WT using 1 × 10^4^ iRBCs per mouse intraperitoneally. Parasitemia was monitored during the course of infection. Each circle represents an individual mouse. Analyses were performed with five animals per group. **B**–**H** Sera collected from infected mice at different time points. Time-dependent changes in serum levels of various cytokines and chemokines as quantified using a bead array (BD). Each circle refers to the response of a mouse, and lines indicate mean values. Statistically significant differences are shown with asterisks (*p < 0.05; **p < 0.01; ***p < 0.001)

To explore the impact of the *Pb*MAP1 on the outcome of blood-stage infection, we infected mice with either WT- or Δ*Pb*MAP1-infected RBCs at 1 × 10^4^ parasites per mouse and characterized the early immune response in WT- or Δ*Pb*MAP1-infected mice. We collected sera on days 3, 5, 7, 10, and 15 after infection and assayed the samples for expression of various cytokines and chemokines (Fig. 10A). The serum levels of IFN-γ were higher in Δ*Pb*MAP1-infected mice than the peak levels in the WT-infected mice, and peak levels were recorded on day 7 after infection (Fig. 10B). Similarly, on day 7 after infection, the levels of TNF-α, IL-6, and IL-10 were higher in ΔPbMAP1-infected mice (Fig. 10C–E). Conversely, on day 7 after infection, the levels of MCP-1 and CCL4 were higher in WT-infected mice than in ΔPbMAP1-infected mice (Fig. 10F, G). Over the infection time, the levels of CCL4 became equivalent in the two groups and continued to increase until day 10 after infection. Similarly, IL-12p70 levels increased with the progression of infection, although no significant differences were noted between the two groups (Fig. 10H).

## Discussion

The invasion of *Plasmodium* into RBCs is a rapid and complex process that involves RBC adhesion, apical reorientation, mobile junction complex, and parasitophorous vacuole membrane (PVM) formation and modification in a series of dynamic steps [24–26]. The key proteins associated with RBC parasite invasion include various proteins located on the merozoite surface, in micronemes, rhoptries, and dense granules [27–29]. When a merozoite invades RBCs, the parasite’s surface proteins bind to RBC surface receptors, increasing calcium concentration in the cytoplasm of the parasite; this in turn stimulates the secretion of microneme proteins, such as TRAP, which plays an important role in the gliding motility and invasion, leading to a series of invasion steps [30–34]. Therefore, adhesion is the first step for *Plasmodium* to invade RBCs, and proteins located on the surface of merozoites play pivotal roles in the process of invasion. However, specific surface proteins that bind to RBCs have not been fully characterized, and these proteins may be important virulence factors in the process of infection.

Glycosaminoglycans (GAGs) are long linear carbohydrate chains that are attached to the core proteins to form proteoglycans. HS is a GAG and comprises alternating glucosamine and uronic acid residues in the repeated disaccharide unit (-4GlcAβ1-4GlcNAcα1-) [6, 14, 35]. HS is localized to the cell surface and in the extracellular matrix of many tissues, and it is implicated in multiple aspects of the *Plasmodium* life cycle [36, 37]. As such, HS is the major receptor for *Plasmodium* invading RBCs [38–41].

Early studies reported that peptides that can bind to heparin contain one or more consistent heparin-binding motifs, namely [-X-B-B-X-B-X-] or [- X-B-B-B-X-X-B-X-], where B is the basic residue of basic amino acids, such as lysine, arginine, and histidine, and X is a hydrophilic amino acid residue [42]. Previous studies on *P. falciparum* indicated that the binding of *Pf*EMP1 to HS receptor on RBCs relies on the presence of multiple heparin-binding motifs in the DBLα region [39]. In the present study, we identified a novel protein, *Pb*MAP1, which contains heparin-binding motifs, indicating that *Pb*MAP1 can potentially bind HS-like receptor. Therefore, the binding of *Pb*MAP1 to the RBCs surface receptor was investigated.

Specifically, our IFA and electron microscopy demonstrated that *Pb*MAP1 is expressed on the surface of *P. berghei*. As a surface protein, *Pb*MAP1 may be involved in the interaction between the parasite and RBC surface receptors. Based on three-dimensional (3D) structural modeling of *Pb*MAP1 and its heparin-binding motifs, we expressed and purified the recombinant protein GST-*Pb*MAP1 in *Escherichia coli* and used the GST protein as the negative control for functional verification. Heparin-binding and competitive inhibition experiments showed that GST-*Pb*MAP1 could bind to heparin in a concentration-dependent manner (Fig. 2). IFA, western blotting, and flow cytometry further indicated that the *Pb*MAP1 protein could bind to the surface of RBCs (Fig. 3). Therefore, *Pb*MAP1 may be involved in binding to HS-like receptor on the RBC surface.

Furthermore, *Pb*MAP1 was knocked out to assess its function. We found that the pathogenicity of the Δ*Pb*MAP1 strain in mice was attenuated compared to that of the WT strain (Fig. 8). The use of *Pb*MAP1-knockout strain further demonstrated the role of the *Pb*MAP1 protein in the binding to RBCs. In addition, we observed that mice immunized with *Pb*MAP1-specific peptides achieved immune protection (Fig. 5). Therefore, *Pb*MAP1 may play important roles in the binding and invasion of *P. berghei* to RBCs. Interestingly, we found that with the continuous replication of the parasites, the infectivity of the Δ*Pb*MAP1 parasite seemed recovered. Therefore, we speculated that some ways of compensation may occur. We collected WT and Δ*Pb*MAP1 strains and searched for genes with significant differences in transcript levels after *Pb*MAP1 knockout using qRT-PCR. The transcript level of *MAEBL* was surprisingly found significantly upregulated, but the transcript levels of 10 other merozoite genes were significantly downregulated. These results confirmed that *Pb*MAP1 knockout affected *MAEBL* expression (Fig. 8).

To assess the potential mechanism through which injection with the Δ*Pb*MAP1 strain can confer immune protection in mice, we examined changes in cytokine levels after immunizing with the Δ*Pb*MAP1 strains. Levels of IFN-γ, TNF-α, IL-6, and IL-10, which are important for the activation of protective immunity, were significantly increased at the early stage of Δ*Pb*MAP1 infection. Therefore, *Pb*MAP1 expression is likely beneficial to the parasite by preventing protective immune responses in the host.

## Conclusion

The present study showed that the surface protein *Pb*MAP1 is involved in the binding of *P. berghei* to the HS receptor on RBC surface. The absence of *Pb*MAP1 is presumably deleterious to the parasite, as it evokes a specific immune response in the host. Our findings revealed the interaction between *Pb*MAP1, a novel *P. berghei* surface protein, and HS-like receptor during invasion. Simultaneously, these findings can be useful approaches for deep characterization of *P. falciparum* proteins in human malaria.

### Supplementary Information

**Additional file 1: Table S1.** Primer sequences referred to in this study.

## Data Availability

The datasets supporting the conclusions of this article are included within the article and its additional file.

## Abbreviations

RBCs: Red blood cells
*Pb*ANKA: *Plasmodium berghei* ANKA
HS: Heparan sulfate
GAGs: Glycosaminoglycans
MSP: Merozoite surface protein
EBL: Erythrocyte binding antigens
MAEBL: Merozoite adhesive erythrocytic binding protein
IP: Intraperitoneal
PBS: Phosphate buffer saline
SDS-PAGE: Sodium dodecyl sulfate-polyacrylamide gel electrophoresis
HS: Heparan sulfate
CSA: Chondroitin sulfate A
RPMI: Roswell Park Memorial Institute
DAPI: 2′,6-diamidino-2-phenylindole
IV: Intravenous
WT: Wildtype
IFN-γ: Interferon gamma
TNF-α: Tumor necrosis factor alpha
IL: Interleukin
MCP-1: Monocyte chemoattractant protein-1
CCL4: Macrophage inflammatory protein-1β

## References

[1] World Health Organization: World malaria report 2021. https://www.who.int/malaria/publications/world-malaria-report-2021/report/en/

[2] Miller LH, Baruch DI, Marsh K, Doumbo OK (2002). The pathogenic basis of malaria. Nature..

[3] Gaur D, Mayer DCG, Miller LH (2004). Parasite ligand–host receptor interactions during invasion of erythrocytes by *Plasmodium* merozoites. Int J Parasitol..

[4] Gilson PR, Nebl T, Vukcevic D, Moritz RL, Sargeant T, Speed TP (2006). Identification and stoichiometry of glycosylphosphatidylinositol-anchored membrane proteins of the human malaria parasite *Plasmodium falciparum*. Mol Cell Proteomics..

[5] Kobayashi K, Kato K, Sugi T, Yamane D, Shimojima M, Tohya Y (2009). Application of retrovirus-mediated expression cloning for receptor screening of a parasite. Anal Biochem..

[6] Zimmermann R, Werner C, Sterling J (2018). Exploring structure–property relationships of GAGs to tailor ECM-mimicking hydrogels. Polymers (Basel)..

[7] Rasti N, Wahlgren M, Chen Q (2004). Molecular aspects of malaria pathogenesis. FEMS Immunol Med Microbiol..

[8] Lima M, Rudd T, Yates E (2017). New applications of heparin and other glycosaminoglycans. Molecules..

[9] Vogt AM, Pettersson F, Moll K, Jonsson C, Normark J, Ribacke U (2006). Release of sequestered malaria parasites upon injection of a glycosaminoglycan. PloS Pathog..

[10] Carruthers VB, Håkansson S, Giddings OK, Sibley LD (2000). *Toxoplasma gondii* uses sulfated proteoglycans for substrate and host cell attachment. Infect Immun..

[11] Boyle MJ, Richards JS, Gilson PR, Chai W, Beeson JG (2010). Interactions with heparin-like molecules during erythrocyte invasion by *Plasmodium falciparum* merozoites. Blood..

[12] Azzouz N, Kamena F, Laurino P, Kikkeri R, Mercier C, Cesbron-Delauw M (2013). *Toxoplasma gondii* secretory proteins bind to sulfated heparin structures. Glycobiology..

[13] Chen Q, Barragan A, Fernandez V, Sundström A, Schlichtherle M, Sahlén A (1998). Identification of Plasmodium falciparum erythrocyte membrane proteins 1 (PfEMP1) as the rosetting ligand of the malaria parasite P. falciparum. J Exp Med..

[14] Vogt AM, Barragan A, Chen Q, Kironde F, Spillmann D, Wahlgren M (2003). Heparan sulfate on endothelial cells mediates the binding of *Plasmodium falciparum*-infected erythrocytes via the DBL1alpha domain of *Pf*EMP1. Blood..

[15] Kobayashi K, Kato K, Sugi T, Takemae H, Pandey K, Gong H (2010). *Plasmodium falciparum* BAEBL binds to heparan sulfate proteoglycans on the human erythrocyte surface. J Biol Chem..

[16] Baum J, Chen L, Healer J, Lopaticki S, Boyle M, Triglia T (2009). Reticulocyte-binding protein homologue 5 – an essential adhesin involved in invasion of human erythrocytes by *Plasmodium falciparum*. Int J Parasitol..

[17] Xiao L, Yang C, Patterson PS, Udhayakumar V, Lal AA (1996). Sulfated polyanions inhibit invasion of erythrocytes by plasmodial merozoites and cytoadherence of endothelial cells to parasitized erythrocytes. Infect Immun..

[18] Sanni LA, Fonseca LF, Langhorne J (2002). Mouse models for erythrocytic-stage malaria. Methods Mol Med..

[19] Aurrecoechea C, Brestelli J, Brunk BP, Dommer J, Fischer S, Gajria B (2009). PlasmoDB: a functional genomic database for malaria parasites. Nucleic Acids Res..

[20] Zhang D, Jiang N, Chen Q (2019). ROP9, MIC3, and SAG2 are heparin-binding proteins in *Toxoplasma gondii* and involved in host cell attachment and invasion. Acta Trop..

[21] Goel VK, Li X, Chen H, Liu S-C, Chishti AH, Oh SS (2003). Band3 is a host receptor binding merozoite surface protein 1 during the *Plasmodium falciparum* invasion of erythrocytes. Proc Natl Acad Sci U S A..

[22] Janse CJ, Ramesar J, Waters AP (2006). High-efficiency transfection and drug selection of genetically transformed blood stages of the rodent malaria parasite *Plasmodium berghei*. Nat Protoc..

[23] Kyes S, Pinches R, Newbold C (2000). A simple RNA analysis method shows var and rif multigene family expression patterns in *Plasmodium falciparum*. Mol Biochem Parasitol..

[24] Gilson PR, Crabb BS (2009). Morphology and kinetics of the three distinct phases of red blood cell invasion by *Plasmodium falciparum* merozoites. Int J Parasitol..

[25] Aikawa M, Miller LH, Johnson J, Rabbege J (1978). Erythrocyte entry by malarial parasites. A moving junction between erythrocyte and parasite. J Cell Biol..

[26] Cowman AF, Crabb BS (2006). Invasion of red blood cells by malaria parasites. Cell..

[27] Sam-Yellowe TY (1996). Rhoptry organelles of the apicomplexa: their role in host cell invasion and intracellular survival. Parasitol Today..

[28] Cowman AF, Tonkin CJ, Tham WH, Duraisingh MT (2017). The molecular basis of erythrocyte invasion by malaria parasites. Cell Host Microbe..

[29] Zhao X, Chang Z, Tu Z, Yu S, Wei X, Zhou J (2014). PfRON3 is an erythrocyte-binding protein and a potential blood-stage vaccine candidate antigen. Malar J..

[30] Sultan AA, Thathy V, Frevert U, Robson KJH, Crisanti A, Nussenzweig V (1997). TRAP is necessary for gliding motility and infectivity of *plasmodium* sporozoites. Cell..

[31] Bargieri DY, Thiberge S, Tay CL, Carey AF, Rantz A, Hischen F (2016). *Plasmodium* merozoite TRAP family protein is essential for vacuole membrane disruption and gamete egress from erythrocytes. Cell Host Microbe..

[32] Buscaglia CA, Coppens I, Hol WGJ, Nussenzweig V (2003). Sites of interaction between aldolase and thrombospondin-related anonymous protein in *plasmodium*. Mol Biol Cell..

[33] Lovett JL, Sibley LD (2003). Intracellular calcium stores in *Toxoplasma gondii* govern invasion of host cells. J Cell Sci..

[34] Billker O, Lourido S, Sibley LD (2009). Calcium-dependent signaling and kinases in apicomplexan parasites. Cell Host Microbe..

[35] Dennissen MABA, Jenniskens GJ, Pieffers M, Versteeg EMM, Petitou M, Veerkamp JH (2002). Large, tissue-regulated domain diversity of heparan sulfates demonstrated by phage display antibodies. J Biol Chem..

[36] McQuaid F, Rowe JA (2020). Rosetting revisited: a critical look at the evidence for host erythrocyte receptors in *Plasmodium falciparum* rosetting. Parasitology..

[37] Adams Y, Kuhnrae P, Higgins MK, Ghumra A, Rowe JA (2014). Rosetting *Plasmodium falciparum*-infected erythrocytes bind to human brain microvascular endothelial cells in vitro, demonstrating a dual adhesion phenotype mediated by distinct P. falciparum erythrocyte membrane protein 1 domains. Infect Immun..

[38] Zhang Y, Jiang N, Lu H, Hou N, Piao X, Cai P (2013). Proteomic analysis of *Plasmodium falciparum* schizonts reveals heparin-binding merozoite proteins. J Proteome Res..

[39] Barragan A, Fernandez V, Chen Q, von Euler A, Wahlgren M, Spillmann D (2000). The Duffy-binding-like domain 1 of *Plasmodium falciparum* erythrocyte membrane protein 1 (PfEMP1) is a heparan sulfate ligand that requires 12 mers for binding. Blood..

[40] Kobayashi K, Kato K (2016). Evaluating the use of heparin for synchronization of in vitro culture of *Plasmodium falciparum*. Parasitol Int..

[41] Weiss GE, Gilson PR, Taechalertpaisarn T, Tham WH, de Jong NWM, Harvey KL (2015). Revealing the sequence and resulting cellular morphology of receptor–ligand interactions during *Plasmodium falciparum* invasion of erythrocytes. PLOS Pathog..

[42] Cardin AD, Weintraub HJ (1989). Molecular modeling of protein–glycosaminoglycan interactions. Arterioscler Dallas Tex..

