# Supplementary material for: A heparin-binding protein of Plasmodium berghei is associated with merozoite invasion of erythrocytes
Source: Parasit Vectors. 2023 Aug 10;16:277. doi: 10.1186/s13071-023-05896-w (PMC10416508; doi:10.1186/s13071-023-05896-w)
Supplement: Supplementary file 1 — Additional file 1: Table S1. Primer sequences referred to in this study. [file 13071_2023_5896_MOESM1_ESM.docx]

Table S1: Primers used in this study:

| Experiment | Primer name | Nucleotide sequence |
| --- | --- | --- |
| WT KO construct | PbKO5'-FW | GGGCCCTGTGCATACTCAATTACCTACGC |
|  | PbKO5'-RV | ATCGATATATTACGATGTGGATTCTTTCACA |
|  | PbKO3'-FW | GGATCCCATTTACGATTTTAGCAGTTTGTAC |
|  | PbKO3'-RV | GCGGCCGCATATGCAGCGACGAAGATTAC |
| WT complement construct | sgRNA | AGATGGAGATGGCTGTCTAGGTTTTAGAGCTAGAAATAGC |
|  | C5UTR-F | GaccatgattacgccaagcttAAGGTGTATTTTTTTTTATACTAGCT  ATAGATG |
|  | C5UTR-R | atacatATATTACGATGTGGATTCTTTCACATT |
|  | C3UTR-F | atATTGGAAGTGGAGGACGGGA |
|  | C3UTR-R | AattgtaaacttaaggaattcAAAATAAAGTCGTCTTAGATAT  AGGTTGTG |
| Intergration-specific PCR analysis | F | GAATACACGCTCATGCTA |
|  | R | TCATCCTCACCCTCACTA |
|  | F1 | TAATCGTCTAAGAGTTTTAAATATA |
|  | R1 | AATTCATACACAAACATACAAAAA |
|  | F2 | GATCCCGTTTTTCTTACTTATATA |
|  | R2 | AAAAATATAGTGGATAATAAGGAAT |
| Integrated complementation-specific PCR analysis | F3 | GATGCATTATCATGCATATTCAC |
|  | R3 | CATAGCTAAACGGTTTGTTATAAGA |
|  | F4 | GATACCTGAAAAATCATATCCTTAT |
|  | R4 | CAGACGATTACTACTTCGATAGTTA |
|  | F5 | GCAGATGATTATAGAATATGGAGTC |
|  | R5 | GTGATCCTGTTGAAGTATAACCTAA |
|  | *Pb*MAP1-2F | GTAAAAACACCCCCAAAGAAA |
|  | *Pb*MAP1-2R | CTTGCTCACATTTTCTAAACTATCA |
| Southern blot | F | GAGAGTAATATTGATATTATGAATA |
|  | R | GCTCTTATGTATATATCATTTATAC |
| qPCR | 142590F | ATTGCATCAGCCAAACCCAC |
|  | 142590R | CGGAACAGTTACGGGAGTACAA |
|  | 113780F | TGCGTTTATGGACCCCCTAC |
|  | 113780R | TAGCAGCATCACGCCAAAGA |
|  | MSP1F | ACGGAACAAGTGCCTGTAGT |
|  | MSP1R | GCTGCTGGTACGGTTGAAGA |
|  | AMA1F | CCCATGTGAACCTACGCAAC |
|  | AMA1R | ACTCTCTGGTTTGATGGGCTT |
|  | RhopH2F | GGCCCCCGTTTGATATTTGC |
|  | RhopH2R | TCGTCACGATTTATGGGTGCT |
|  | actinF | TATTCATCAGGCCGTACAACAGG |
|  | actinR | AGCCAAATCTAATCTCATTATTGCAT |
|  | 1024600F | GTGCCCCTGGCGATCTTAAT |
|  | 1024600R | TTAAACGTTTCAGGGGGCGA |
|  | 0712100F | CAAAAGCTGGGGGAGTTGGA |
|  | 0712100R | CTGTGTACTCTGTCTTCGGCT |
|  | 0211900F | GAGCGATGCGCAAACTACTG |
|  | 0211900R | AAAGGGAATTTGGGGGAGCA |
|  | 0418000F | TTGAGGAGCACCCTTCTTCAAA |
|  | 0418000R | TAAAGAGGGGCCAAGAAAGGG |
|  | 1228100F | GAGGTCGAGGTCGAGGAAGA |
|  | 1228100R | GGTTTTCTGCCTCGTTTTGCT |
|  | 1212700F | TTCTCAGCACCCCGCTATTC |
|  | 1212700R | CCTAAAATACCCTCGTTGACGGA |
|  | 0618200F | ACAATAGTAGTGGCGAACAAGA |
|  | 0618200R | AGGCTTCCCGATTCTATGCT |
|  | 0610100F | TTAAGAGCGCCCCACCCTAT |
|  | 0610100R | GCGCAATATCTTCTGCTGCTC |
|  | 0103300F | GCACATGAATAAAAAGGCGATGGA |
|  | 0103300R | TCATGTCCGCTGTTTGGAGT |
|  | 0112100F | AAGCATGGAGAGCTGAATGGAA |
|  | 0112100R | TCCGCGACTAAGCATTCACC |
|  | 1450600F | ATCGATATGTATCAAGCACAATGGA |
|  | 1450600R | ACAGGGTTATATGCCTCATTTTGT |
|  | 1450300F | TAACAGATCCTGCACCAGCC |
|  | 1450300R | CAATGGATCGACAGCTGGGT |
|  | 1102200F | ACCTGCAGATGTATTCCAGGATTTT |
|  | 1102200R | TGCATTTACATCACATCCGCCA |
|  | 1031300F | GATTCTCGGGACTATCCTTCCT |
|  | 1031300R | TCATTCATTGCCATTTGGGGAA |
|  | 1134600F | AATAATTTGTGGAAGCAGTTCAGC |
|  | 1134600R | TTATAGAGGAAGCTTGTATTCGCAA |
|  | 1358100F | TGTTTCTGTATTCCCGTTTCCT |
|  | 1358100R | GCACCGCCTAGTAAACCAAA |
|  | 0304700F | CTAAGACAGTAGACCCGCCAA |
|  | 0304700R | GGATCTCTGGAGGGAGGGT |
|  | 0304800F | ATGCTGACCATGCAGCAACTA |
|  | 0304800R | ATGTTGGGGGCCATATATATCAACT |
|  | 0304900F | TAATGCCCAAAGCACACCCA |
|  | 0304900R | GCATTTGCAGAGGCAGTGTT |
|  | 0901300F | ATTCCTTGGTTTGCTTCTTGCT |
|  | 0901300R | CTGCAATCCACACACCCCAA |
|  | 0933500F | AGAAGATGGAAAACAAGCCAACA |
|  | 0933500R | GCATGCACGAGGAACAGCAA |
|  | 0309500F | TTGGTCGGATTGGACACCAT |
|  | 0309500R | ATTCACACGATCGAGTTTCCTCT |
|  | 0504100F | GCCATCATTAAAAGAAGAAATCCCC |
|  | 0504100R | GCGCTTTTTCACACACCACT |
|  | 1349100F | GGGTATGAGCGAGGACGATT |
|  | 1349100R | CACCGTTTGCATCTGAACCC |
|  | 1321700F | AGGTATGTGTGGGTCTTGCTG |
|  | 1321700R | ATGACCGCCATCACATCCAA |
|  | 1107100F | GTCAAGTTTCAGCACCAGGC |
|  | 1107100R | TCCAGCAACATGAGGAGCAG |
|  | 0713100F | AGGAAACTTTTCGGCAGCAAT |
|  | 0713100R | TGGACAGCGTCAGCCTTTTT |
|  | 0932000F | TGAACACAGTGCAGAAGGGG |
|  | 0932000R | TGAGCTTGAACTTGTCGTATCACT |
|  | 1315700F | TCTCTTGTACCCCCACCCAA |
|  | 1315700R | AAACCAGCCATAGGGGCAAA |
|  | 0911700F | AGCTGCAGCAGGAAATGAAAA |
|  | 0911700R | TGTGCACACTGTTAGCTCCAT |
|  | 1443300F | TGCTTAACTACCACCAATCCTGT |
|  | 1443300R | TGCATAGACTCATCTGTTATGGCT |
|  | 0314200F | GTGAAGAGAGGCTAAGGAGGG |
|  | 0314200R | GGTCAGCCTCCCCTAAGACA |
|  | 1424400F | CCCAAAATGCGGCACAAAGA |
|  | 1424400R | TTTCCGTCCTTCCTCCGTTC |
|  | 1424600F | TGTCATCAGCTAACCAATTAGGAA |
|  | 1424600R | AAAGTTGATTTTCCATAACCGCA |
|  | 1425000F | GAGTCAGAAAACATGTTAGACGGG |
|  | 1425000R | CAGTTTCTCTTCTTCCTCGCCT |
|  | 1425200F | ACTGAGCAAGGAGTAGACACA |
|  | 1425200R | CATTAACCGATATCCATTTCCACA |
|  | 1425350F | AACGTAGTGGGTGCACAAGG |
|  | 1425350R | CCCCAATTCCTTCAGCATAACC |
|  | 1425500F | ACCAAATCAGGAGAGTGAAACC |
|  | 1425500R | GGCGTAAAAGTTACTCGTTGGT |
|  | 1433900F | TGGTATGCCAATTTGCGAGT |
|  | 1433900R | TTTTACACTTGCGTTATTTTCTCCA |
|  | 1433500F | GTGAAATTGTGGTCTCCGCT |
|  | 1433500R | TCGTTTGGAGGCAAACACATT |
|  | 1432900F | TTTGAGGTTGTAGATACGGGGG |
|  | 1432900R | TGCACGCCAGCTATACCATC |
|  | 1432500F | AGGCGTTGAATTGAGTGAAAAAGA |
|  | 1432500R | TCATTTTGTTCTGGGTTCCCCT |
|  | 1432400F | TCCAGAGCATTCATGCAACCA |
|  | 1432400R | CCCGCAGAAGCTGAAAAAGA |
|  | 1431900F | GAAGAAATGCCAGATGCGCTG |
|  | 1431900R | TTCCCTGTAACTTTTGGCCATTC |
|  | 1431700F | AGCTTGGTTACCGAGTCAAAA |
|  | 1431700R | CCTACTGCCAACCCTGAACC |
|  | 1431600F | GTGCAGGACCCCATGCTATATT |
|  | 1431600R | TCTCCATTTCCCCATCCTCGT |
|  | 1431500F | TCAGATATCCCATCAAATGCGTG |
|  | 1431500R | GCCATTTCCTATGTAAATTGAAGCA |
|  | 1430900F | ACAGGTAATAATAGACGTCACGCA |
|  | 1430900R | TCCTCATTTTCATGGGGGTCA |
|  | 1429900F | CAGCAGAATTTTGGACACCTTT |
|  | 1429900R | ACGTTACACACAACTCGTTCA |
|  | 1429300F | AGGTTTATGCTATTTGGAACGGC |
|  | 1429300R | ACGTTGCTCCAGGAAGATCAC |
|  | 1429000F | ACTAAATCCCCAATCACAAAATCAA |
|  | 1428800F | GAATTGCTCAGTGTGCTGCC |
|  | 1428700F | AATCGTCCGCCGATGATGAA |
|  | 1428700R | TCCGGACCAGGTTTTTCTATCC |
|  | 1428300F | ACAAACATACCAGAACTTCTCCAC |
|  | 1428300R | TGTCAACCACTTGAACCCACA |
|  | 1428100F | ATCCAAGGAATTTGGAACCACC |
|  | 1428100R | GGGCTTTGTTCATCATCATTTGT |
|  | 1427800F | ATAGCACACGCTGTAAGCAC |
|  | 1427800R | ATCCTTTGGGGCCCATTTTT |
|  | 1427700F | GCGGTTTGGGTTTAGATGAGG |
|  | 1427700R | CGCCATTTGTGAGGTTGCC |
|  | 1427400F | TACCCTACCCAAAAATCAGTTCA |
|  | 1427400R | TGTCACATATCTTGAGGGTCGT |
|  | 1427200F | AATCAGGGGGCCAAGTTTTGA |
|  | 1427200R | GTGTCTCCAATAGCCCGTGA |
|  | 1427000F | ACACTTCCAATACCTGACCACTT |
|  | 1427000R | ACGCATGTTGCTCCATCTTT |
|  | 1426600F | ATGCCGACTGGTGTGAGTG |
|  | 1426600R | TCTTTGTCTCTTGGTTCAGATCTAT |
|  | 1426400F | GTGCAAATGAAGCATTTTGGGT |
|  | 1426400R | ACAGTTCCTTAACAAATGCGTGG |
|  | 1425700F | AAACGACATCATACGATCAAAAATG |
|  | 1425700R | TGCAAGATGAGAGAAAGTACAAAAA |
|  | 1426000F | TCATATAGCGAAAGCATGAAAAGT |
|  | 1426000R | AAGCGTTTGTTTCCAATGTGT |
|  | 0201800F | TTGAACAAGTTGGCGGAGTT |
|  | 0201800R | TTTTAAAGAACAGCCATTTACTCCT |
|  | 0926200F | AGCGAATTTTCAAAACCCGAA |
|  | 0926200R | ACTTTCATCAGGTAGAAATGCAAA |
|  | 0416000F | TCATGTGGAGCCGCAGTTTT |
|  | 0416000R  1332700F  1332700R | AAACGGACTAGACGGGTTAGA  TGCCCCAAAAGTCAAACCCA  GCTCCCTTTTTGAAAGCAGGT |
